# Persistent sexual arousal syndrome and schizoaffective disorder: a case report

**DOI:** 10.1192/j.eurpsy.2025.2316

**Published:** 2025-08-26

**Authors:** M. J. Muñoz Algar, P. Bernal García

**Affiliations:** 1 PSICHIATRY, HOSPITAL DR. R. LAFORA, MADRID, Spain

## Abstract

**Introduction:**

Persistent sexual arousal syndrome (PSAS) is characterized by unwanted and distressing genital sensations that persist for long time periods without concurrent sexual desire or fantasies. The aetiology of this remains largely enigmatic although it is likely that the condition has a diverse set of neurological, vascular, pharmacological, and psychological precipitants.

**Objectives:**

To analyze the case of a woman with schizoaffective disorder (severe depressive decompensation) and a comorbid PSAS.

**Methods:**

We study the clinical case of a 47-year-old patient who was admitted to the acute care unit due to a major depressive condition with psychotic symptoms in the context of a schizoaffective disorder after partial abandonment and erratic taking of the medication she was previously taking.

Before the admission, the patient was hypomimic, perplexed, with psychomotor inhibition, bradypsychia, thought blockages, sadness, emotional lability, apathy, anhedonia, paranoidism, phenomena of reading and thought control, self-referentiality, delusional ideas of harm and auditory pseudohallucinations in the form of voices that urge her to harm herself. In addition, the patient presented several “sexual” crises that appeared paroxysmal throughout the day, consisting of episodes of sexual hyperarousal in the absence of desire, experienced with intense guilt. Initially, a differential diagnosis was made through an extensive history and organic screening, and she was finally diagnosed with a comorbid PSAS.

**Results:**

Complementary tests (complete blood, urine and imaging tests) were normal.

At the pharmacological level, several strategies were used that were ineffective: paliperidone up to 18mg/day that had to be withdrawn due to intolerable extrapyramidal effects, olanzapine up to 15mg/day with high sleepiness and finally caripracin up to 12mg/day with good tolerance and efficacy. Stabilizing treatment (valproic acid 1000mg/day with optimal blood levels (99.3 microgr/mL)) were added. However, after a month and a half of admission and given the little improvement of the depressive symptoms, even having added an SSRI for 2 weeks at full doses, it was decided together with the patient and her family to start Electroconvulsive Therapy (ECT)sessions. The patient received 12 sessions of bitemporal ECT with onset of response from the 6th session, with a complete remission of the sexual crisis and depressive symptoms.

**Conclusions:**

To our knowledge, Yero et al., reported the first two cases of patients with concomitant PSAS and bipolar disorder treated with ECT. It is important to understand how sexual symptoms differ in PSAS and bipolar disorder. Remission of the mood episode could have been accompanied by resolution of the sexual symptoms. Although ECT was successful, the mechanism of action in treating PSAS is unknown, and it is premature to suggest that it should be recommended as a first line treatment of PSAS.

**Disclosure of Interest:**

None Declared

